# Antibody-Dependent Enhancement Infection Facilitates Dengue Virus-Regulated Signaling of IL-10 Production in Monocytes

**DOI:** 10.1371/journal.pntd.0003320

**Published:** 2014-11-20

**Authors:** Tsung-Ting Tsai, Yi-Jui Chuang, Yee-Shin Lin, Chih-Peng Chang, Shu-Wen Wan, Sheng-Hsiang Lin, Chia-Ling Chen, Chiou-Feng Lin

**Affiliations:** 1 Institute of Basic Medical Sciences, College of Medicine, National Cheng Kung University, Tainan, Taiwan; 2 Department of Microbiology and Immunology, College of Medicine, National Cheng Kung University, Tainan, Taiwan; 3 Center of Infectious Disease and Signaling Research, National Cheng Kung University, Tainan, Taiwan; 4 Institute of Clinical Medicine, College of Medicine, National Cheng Kung University, Tainan, Taiwan; 5 Center of Translational Medicine, Taipei Medical University, Taipei, Taiwan; 6 Graduate Institute of Medical Sciences, College of Medicine, Taipei Medical University, Taipei, Taiwan; 7 Department of Microbiology and Immunology, College of Medicine, Taipei Medical University, Taipei, Taiwan; University of North Carolina at Chapel Hill, United States of America

## Abstract

**Background:**

Interleukin (IL)-10 levels are increased in dengue virus (DENV)-infected patients with severe disorders. A hypothetical intrinsic pathway has been proposed for the IL-10 response during antibody-dependent enhancement (ADE) of DENV infection; however, the mechanisms of IL-10 regulation remain unclear.

**Principle Finding:**

We found that DENV infection and/or attachment was sufficient to induce increased expression of IL-10 and its downstream regulator suppressor of cytokine signaling 3 in human monocytic THP-1 cells and human peripheral blood monocytes. IL-10 production was controlled by activation of cyclic adenosine monophosphate response element-binding (CREB), primarily through protein kinase A (PKA)- and phosphoinositide 3-kinase (PI3K)/PKB-regulated pathways, with PKA activation acting upstream of PI3K/PKB. DENV infection also caused glycogen synthase kinase (GSK)-3β inactivation in a PKA/PI3K/PKB-regulated manner, and inhibition of GSK-3β significantly increased DENV-induced IL-10 production following CREB activation. Pharmacological inhibition of spleen tyrosine kinase (Syk) activity significantly decreased DENV-induced IL-10 production, whereas silencing Syk-associated C-type lectin domain family 5 member A caused a partial inhibition. ADE of DENV infection greatly increased IL-10 expression by enhancing Syk-regulated PI3K/PKB/GSK-3β/CREB signaling. We also found that viral load, but not serotype, affected the IL-10 response. Finally, modulation of IL-10 expression could affect DENV replication.

**Significance:**

These results demonstrate that, in monocytes, IL-10 production is regulated by ADE through both an extrinsic and an intrinsic pathway, all involving a Syk-regulated PI3K/PKB/GSK-3β/CREB pathway, and both of which impact viral replication.

## Author Summary

IL-10 has multiple cellular functions, including anti-inflammatory and immunomodulatory effects. Clinical studies have demonstrated that the serum levels of IL-10 are significantly increased in DENV-infected patients with severe disorders. However, the molecular mechanism underlying DENV-induced IL-10 production is still unresolved. In this study, we demonstrate a molecular mechanism for DENV-induced IL-10 production, which may be exacerbated by ADE through Fcγ receptor-mediated extrinsic and intrinsic pathways, leading to IL-10/SOCS3-mediated advantages for viral replication. With or without Fcγ receptor- or CLEC5A-mediated DENV infection, a common Syk/PKA-regulated PI3K/PKB activation results in a decrease in GSK-3β activity followed by an increase in CREB-mediated IL-10 expression not only in THP-1 monocytic cells but also in human monocytes. Taken together, we demonstrate a potential regulation and a pathological role for ADE-induced IL-10 overproduction during DENV replication. Therefore, inhibiting immunosuppression by targeting the IL-10 pathways identified in this study may help to prevent the progression of severe dengue diseases.

## Introduction

Four serotypes of *dengue virus* (DENV) – a mosquito-borne human pathogen belonging to the family *Flaviviridae* and the genus *Flavivirus* – infect an estimated 50 million people annually and cause a spectrum of illnesses, ranging from mild dengue fever (DF) to the more severe dengue hemorrhagic fever (DHF) and dengue shock syndrome (DSS) [1]. However, it is unclear which antiviral strategies are most appropriate for treating DENV progression, as many aspects of DENV pathogenesis remain controversial, including viral load, virulence, cytotoxicity, the nature of the immune response, autoimmunity [2], [3], and the potential effects of common diseases such as allergies, diabetes, and hypertension [4], [5]. There are no licensed antiviral drugs for DENV treatment. Administration of chloroquine (a 9-aminoquinoline) exerts direct antiviral effects by inhibiting the pH-dependent steps of flavivirus replication, although this drug is failed to inhibit the duration of viremia and antigenemia in DENV patients [6]. Balapiravir (4'-azidocytidine) is developed for the treatment of chronic hepatitis C Virus infection by a nucleoside analogue of RNA-dependent RNA polymerase; however, this drug does not alter the kinetics of viremia and NS1 antigenemia in DENV patients [7]. During the early acute phase of DENV infection, oral prednisolone is not related to prolongation of viremia or other pathogenic effects [8]. A recent trial showing that the α-glucosidase inhibitor celgosivir (6-O butanoyl prodrug of castanospermine) has antiviral activity by modulating the host's unfolded protein response, but it does not significantly reduce viral load or fever burden in DENV patients [9]. The development of a DENV vaccine would represent a powerful new tool for preventing DENV infection. Although a safe vaccine is not yet available, a number of candidate vaccines and strategies for strengthening vaccine efficiency are under active investigation [1], [10], [11].

DENV is an enveloped, single-stranded RNA virus that contains several types of structural proteins, including envelope protein (E), precursor membrane protein, and capsid protein, as well as several types of nonstructural (NS) proteins, including NS1, NS2A, NS2B, NS3, NS4A, NS4B, and NS5 [12]. All of the DENV proteins function in the viral biology and pathogenesis. The DENV E protein is the viral receptor for cell binding and fusion [13]. The cellular targets of DENV include monocytes/macrophages, dendritic cells, B cells, T cells, basophil/mast cells, endothelial cells, epithelial cells, and hepatocytes [14]. DENV infects and/or interacts with cells through a variety of cell-surface molecules, including heparan sulfate [15], integrins [16], dendritic cell-specific intracellular adhesion molecule 3 grabbing non-integrin (DC-SIGN) [17], C-type lectin domain family 5 member A (CLEC5A) [18], and heat shock proteins [19]. An alternative route for DENV infection is receptor-mediated endocytosis, following viral-cell receptor interaction [14]. The generation of antibodies (Abs) against the DENV E protein is fundamental for the host defense; however, such immune responses may increase the risk of developing DHF/DSS upon re-infection, primarily due to the canonical effects of antibody-dependent enhancement (ADE) – a phenomenon in which non-neutralizing anti-E Abs cross-react with the heterogeneous serotypes of DENV and facilitate their binding with Fcγ receptor-bearing cells to cause severe infection [20]. Combined with DENV-induced autoimmunity [2], [3], these effects could represent the primary challenges to DENV vaccine development.

DENV pathogenesis can be affected by many viral factors, including viral load, serotype, and virulence [21], [22]. However, the immunopathogenesis of DENV infection is caused by host-specific immune responses, including immune cell activation (CD4 positive T cells), cytokines (interleukin (IL)-1β, IL-2, IL-6, IL-10, IL-13, IL-18, macrophage migration inhibitory factor, tumor growth factor-β, tumor necrosis factor-α, and interferon (IFN)), chemokines (IL-8, monocyte chemoattractant protein-1, and regulated and normal T cell expressed and secreted), complement activation (C5a and C5b-9), inflammatory mediators (high mobility group box 1), and autoimmunity (auto-Abs against platelets, endothelial cells, and coagulants), all of which have been identified as hallmarks of DHF/DSS [1]–[3], [13], [20], [23], [24]. IL-10 has been proposed to play a role in DENV pathogenesis due to its immunosuppressive functions during IFN resistance and persistent viral infection. Epidemiological studies have demonstrated that IL-10 serum levels are higher in DHF/DSS patients than in DF patients with acute illness [25]–[31]. However, following ADE of DENV infection in monocytes [20], [32]–[34], it has been speculated that the Fcγ receptor might facilitate the IL-10 response to induce the expression of the suppressor of cytokine signaling (SOCS) 3, a downstream effector of IL-10 that mediates immunosuppression [35]. The existence of an intrinsic model for ADE [20], [33], differing from the canonical extrinsic ADE pathway, enhances DENV infection by facilitating IL-10/SOCS3-mediated benefits to escape from antiviral IFN responses, such as type I IFNs production [34] and T cell activation [36]. In this study, we investigate the molecular regulation of IL-10 production in monocytes infected with DENV, both directly and via ADE.

## Materials and Methods

### Antibodies and reagents

The reagents and antibodies used were polybrene, PKA inhibitor H-89, PI3K inhibitor 2-(4-Morpholinyl)-8-phenyl-4H-1-benzopyran-4-one hydrochloride (LY294002), and PKC inhibitor bisindolylmaleimide (Bis) (Calbiochem, San Diego, CA); 4,6-diamidino-2-phenylindole (DAPI), GSK-3 inhibitor BIO, heparin lyase III, chondroitin ABC lyase, O-linked glycosylation inhibitor benzyl-α-GalNAc, N-linked glycosylation inhibitor tunicamycin, Syk inhibitor BAY61-3606, dimethyl sulfoxide (DMSO), and mouse mAb specific for β-actin (Sigma-Aldrich, St. Louis, MO); recombinant human IL-10 (PeproTech, Rocky Hill, NJ); Abs against DENV NS4B and E (GeneTex, San Antonio, TX); Abs against SOCS3, phospho-CREB at Ser133, CREB, phospho-PKB at Ser473, PKB, phospho-GSK-3β at Ser9, GSK-3β, β-catenin, and Mcl-1 (Cell Signaling Technology, Beverly, MA); Abs against TIM1, Axl, and IL-10 (R&D Systems, Minneapolis, MN); Abs against isotype control IgG (Millipore, Billerica, MA); donkey anti-goat IgG conjugated with horseradish peroxidase (HRP) (Santa Cruz Biotechnology, Santa Cruz, CA) and goat anti-rabbit IgG conjugated with HRP (Chemicon International, Temecula, CA); rabbit anti-mouse IgG conjugated with HRP (Abcam, Cambridge, MA); and Alexa Fluor 488- and Alexa Fluor 594-conjugated goat anti-mouse and goat anti-rabbit (Invitrogen, Carlsbad, CA). All drug treatments were assessed for cytotoxic effects using cytotoxicity assays prior to experiments. Non-cytotoxic dosages were used in this study.

### Cell culture and virus strains

Human monocytic THP-1 cells were routinely grown on plastic in RPMI Medium 1640 (RPMI; Invitrogen Life Technologies, Rockville, MD), with L-glutamine and supplemented with 10% heat-inactivated fetal bovine serum (FBS; Invitrogen Life Technologies), 50 units of penicillin, and 50 µg/ml of streptomycin. Baby hamster kidney (BHK) cells and C6/36 cells were cultured in Dulbecco's modified Eagles medium (DMEM; Invitrogen Life Technologies) containing FBS. Monocyte-enriched peripheral blood mononuclear cells (PBMC) were isolated from healthy volunteers by density-gradient centrifugation using Ficoll-paque Plus (GE Healthcare, Piscataway, NJ), washed twice with red blood cell lysis buffer (eBioscience, San Diego, CA), resuspended in RPMI 1640 medium supplemented with 10% heat-inactivated fetal bovine serum, and maintained at 37°C in an atmosphere containing 5% CO_2_ while allowing adherence on uncoated polystyrene flasks during 90 min for monocyte enrichment. Non-adherent cells were gently removed by washing, after which the adherent cells were collected to perform the DENV infection experiment [37]. The protocols and procedures were approved by the institutional review board of the National Cheng Kung University Hospital (No. A-ER-102-123). Four serotypes of DENV (DENV1 8700828, DENV2 PL046 and 454009A, DENV3 8700829A, and DENV4 59201818) were maintained in C6/36 cells. Monolayers of C6/36 cells were incubated with DENV at a MOI of 0.01 and incubated at 28°C in 5% CO2 for 5 days. The virus supernatant was further filtered with 0.22 µm filter, and then stored at −80°C until use. Virus titer was determined by plaque assay, using the BHK cell line.

### DENV infection

Cells were resuspended at a concentration of 5×10^5^ cells/ml in appropriate medium with DENV (MOI  = 1) and incubated for 90 min at 37°C. Then, the cells were washed once with RPMI medium, resuspended at a concentration of 5×10^5^ cells/ml, and incubated at 37°C with 5% CO_2_. Monoclonal anti-E 50-2 IgG_1_ Ab, which can recognize viral E protein and shows both neutralization and enhancement activity, was used to induce ADE of the DENV infection, as described previously [38]. To prepare the UV-inactivated virus, DENV was exposed to a 15 W UV lamp at a distance of 10 cm for 1.5 h. The viral supernatants were checked using plaque assays.

### Plaque assay

BHK-21 cells were plated into 12-well plates (2×10^5^ cells/well) and cultured in DMEM under CO_2_-enriched conditions. After adsorption with a serially diluted virus solution for 1 h, the solution was replaced with fresh DMEM containing 2% FBS and 0.5% methyl cellulose (Sigma-Aldrich). Five days post-infection, the medium was removed, and the cells were fixed and stained with crystal violet solution containing of 1% crystal violet, 0.64% NaCl, and 2% formalin.

### Western blotting

Both procedures are described elsewhere [39]. In brief, total cell lysates were extracted and proteins were separated using SDS-polyacrylamide gel electrophoresis and then transferred to a polyvinylidene difluoride membrane (Millipore). After blocking, blots were developed with the indicated Abs and developed using an ECL Western blot detection kit (Pierce Chemical, Rockford, IL), according to the manufacturer's instructions. Following densitometer-based quantification and analysis using ImageJ software (http://rsbweb.nih.gov/ij/), the relative densities of the identified proteins were calculated.

### IL-10 expression

After treatment, we used a commercial ELISA kit (88-7106-77, eBioscience) to detect the concentration of human IL-10 in cell-conditioned culture medium, according to the manufacturer's instructions. For the luciferase reporter assay, the cells were transiently co-transfected using a GeneJammer reagent (Stratagene, La Jolla, CA), with an IL-10 promoter-driven luciferase reporter (0.2 µg), kindly provided by Dr. Yu-Ming Wang, Institute of Bioinformatics and Biosignal Transduction, National Cheng Kung University, and 0.01 µg of *Renilla* luciferase-expressing plasmid (pRL-TK; Promega, Madison, WI). Twenty-four hours after the transfection, the cells were infected with DENV for 24 h, lysed, and then harvested for luciferase and *Renilla* measurement, using a luciferase assay system (Dual-Glo; Promega). For each lysate, the firefly luciferase activity was normalized to the *Renilla* luciferase activity to assess transfection efficiencies.

### Activity assay

After DENV infection, we used a commercial ProFluor® PKA Assay kit (V1240, Promega) and a PIP3 Mass ELISA kit (K-2500s, Echelon Biosciences, Salt Lake City, UT) to detect the activity of PKA and PI3K in THP-1 cells, according to the manufacturers' instructions.

### Immunostaining

To detect expression of CREB and DENV2 E protein, cells were fixed with 4% paraformaldehyde, permeabilized with 0.5% Triton X-100, and washed twice with ice-cold PBS. Cells were stained with anti-CREB and DENV E Abs, and then with Alexa 488-conjugated goat anti-mouse IgG and Alexa 594-conjugated goat anti-rabbit IgG. DAPI (5 µg/ml) was used for nuclear staining. Cells were visualized under a fluorescent microscope (BX51; Olympus, Tokyo, Japan) or a laser-scanning confocal microscope (SPII; Leica Mikrosysteme Vertrieb, Bensheim, Germany). The three-dimensional images reconstructed from a series of confocal images, along with the z-axis of the cells and the analysis of z-stacks, were reconstructed using the Leica Confocal Software. For flow cytometric analysis, cells were fixed and stained with anti-TIM1, Axl, and NS4B Abs as described elsewhere [40], and then incubated with a mixture of Alexa Fluor 488-conjugated secondary Ab. Cells were analyzed using flow cytometry (FACSCalibur; BD Biosciences, San Jose, CA) with excitation set at 488 nm; emission was detected with the FL-1 channel (515–545 nm). Samples were analyzed using CellQuest Pro 4.0.2 software (BD Biosciences). Small cell debris was excluded by gating on a forward scatter plot.

### RNA interference

Protein was downregulated using lentiviral expression of short hairpin RNA (shRNA) targeting IL-10 (TRCN0000058462 containing the following respective shRNA target sequences: 5'-GCCTACATGACAATGAAGATA-3'), GSK-3β (TRCN0000010551 containing the following respective shRNA target sequences: 5'- CACTGGTCACGTTTGGAAAGA-3'), and a negative control construct (luciferase shRNA, shLuc). The shRNA clones were obtained from the National RNAi Core Facility, Institute of Molecular Biology/Genomic Research Center, Academia Sinica, Taipei, Taiwan. Lentiviruses were prepared and cells were infected according to previously described protocols [39]. In brief, THP-1 cells were transduced by lentivirus, with an appropriate multiplicity of infection, in complete growth medium supplemented with polybrene (Sigma-Aldrich). After transduction for 24 h and puromycin (Calbiochem) selection for 6 days, protein expression was monitored using Western blot analysis. CREB and CLEC5A expression was silenced using commercialized siRNA (clone #1, CREB1-HSS102262 containing the following respective siRNA target sequences: 5′-UUACGGUGGGAGCAGAUGAUGUUGC-3′ and 5′-GCAACAUCAUCUGCUCCCACCGUAA-3′; clone #2, CREB1-HSS102264 containing the following respective siRNA target sequences: 5′-UUGCUGGGCACUAAGAUCUGCUGUC-3′ and 5′-GACAGCAGAUCUUAGUGCCCAGCAA-3′ for CREB silencing and CLEC5A-HSS119041 containing the following respective siRNA target sequences: 5′-AAUAAGCCCAGAGAUGAUCAUGUGC -3′ and 5′-GCACAUGAUCAUCUCUGGGCUUAUU-3′ for CLEC5A silencing) (Invitrogen). Transfection was performed by electroporation using a pipette-type microporator (Microporator system; Digital Bio Technology, Suwon, Korea). After transfection, THP-1 cells were incubated for 18 h in RPMI medium at 37°C before infection. A nonspecific scrambled siRNA kit (StealthTM RNAi Negative Control Duplexes, 12935-100; Invitrogen) was the negative control.

### Statistical analysis

Data obtained from three independent experiments are presented as the mean ± standard deviation (SD). Statistical analysis of data analyses were performed using Prism version 5 (GraphPad Software, San Diego, CA). Two sets of the data were analyzed by an unpaired Student's *t* test. Three or more sets of data were analyzed by one-way ANOVA with Tukey's multiple-comparison posttest. Statistical significance was set at *P*<0.05.

## Results

### DENV infection induces IL-10 production and expression of the downstream effector SOCS3 in THP-1 monocytic cells

**Figure 1:**
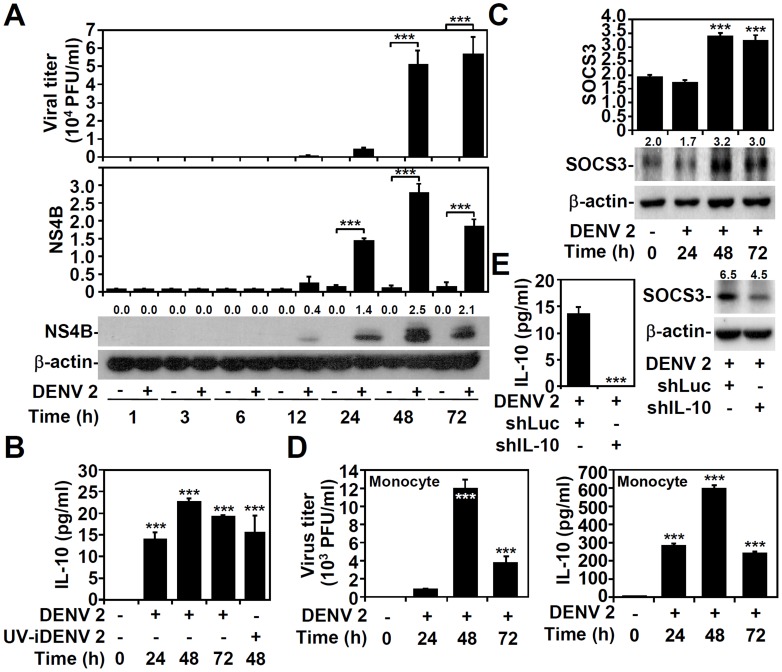
DENV infection induces IL-10 production and activation in monocytes. A: THP-1 cells infected with DENV serotype 2 PL046 (DENV 2, MOI  = 1) were assessed for viral replication by a plaque assay and for the time-kinetic expression of DENV NS4B protein by Western blot analysis. *** P<0.001. B: After the cell-culture supernatants were collected, ELISA was used to quantify IL-10 production in DENV 2 (MOI  = 1)-infected or UV-inactivated DENV 2 (UV-iDENV 2)-treated THP-1 cells. *** P<0.001, compared with untreated cells. C: A Western blot showing the time-kinetic SOCS3 expression in DENV 2 (MOI  = 1)-infected THP-1 cells. *** P<0.001, compared with untreated cells. D: Plaque assays and ELISA were used to quantify viral replication and IL-10 production, respectively, in DENV 2 (MOI  = 1)-infected human peripheral blood monocytes. *** P<0.001, compared with untreated cells. E: IL-10 expression was silenced in THP-1 cells with a lentiviral-based shRNA (shIL-10); luciferase shRNA (shLuc) was used as a negative control. Transfected THP-1 cells were infected with DENV 2 (MOI  = 1) for 48 h, and ELISA and Western blotting were used to detect the expression of IL-10 and SOCS3, respectively. ***P<0.001, compared with control group. For Western blotting results, β-actin was the internal control. One set of representative data obtained from three independent experiments is shown. Following densitometer-based quantification and analysis by using ImageJ software, the relative densities of NS4B and SOCS3 are shown. For all experiments, the quantitative data shown represent mean ± SD values of three independent experiments.

DENV has a variety of cellular targets, the most common being mononuclear phagocytes [14], [41]. Furthermore, IL-10 production is upregulated in monocytes following ADE of DENV infection [20], [32]–[34]. Human monocytic THP-1 cells were infected with DENV serotype 2 PL046, as demonstrated by plaque assays (Figure 1A, upper panel), and the quantitative data of Western blotting revealed the time-kinetic expression of viral NS4B protein (Figure 1A, middle and lower panels), which was first detectable 12 h post-infection and increased significantly (*P<*0.001) by 48 h post-infection. ELISA showed that either DENV infection alone or treatment of ultraviolet-inactivated DENV was sufficient to significantly increase IL-10 production in THP-1 cells (*P<*0.001) (Figure 1B), as well as the significant (*P<*0.001) expression of its downstream target SOCS3 as determined by Western blotting (Figure 1C). Without DENV infection, supernatants of C6/36 cells did not cause IL-10 production (Figure S1 in Text S1). In human peripheral blood monocytes, DENV infection also significantly (*P*<0.001) caused viral replication (Figure 1D, left panel) and IL-10 production (Figure 1D), right panel. To further demonstrate the essential role of IL-10 in SOCS3 expression, a lentiviral-based short hairpin RNA (shRNA) approach was used. In IL-10 silenced cells, the DENV-induced IL-10 expression was significantly abolished (*P*<0.001) (Figure 1E, left panel), accompanied by a decrease in DENV-induced SOCS3 expression (Figure 1E, right panel). These results demonstrate that DENV can induce an IL-10 response in monocytes through an infectious process and/or attachment.

### DENV infection induces protein kinase A (PKA)- and phosphoinositide (PI) 3-Kinase/PKB-regulated cyclic adenosine monophosphate response element-binding (CREB) activation, followed by CREB-mediated IL-10 production

**Figure 2:**
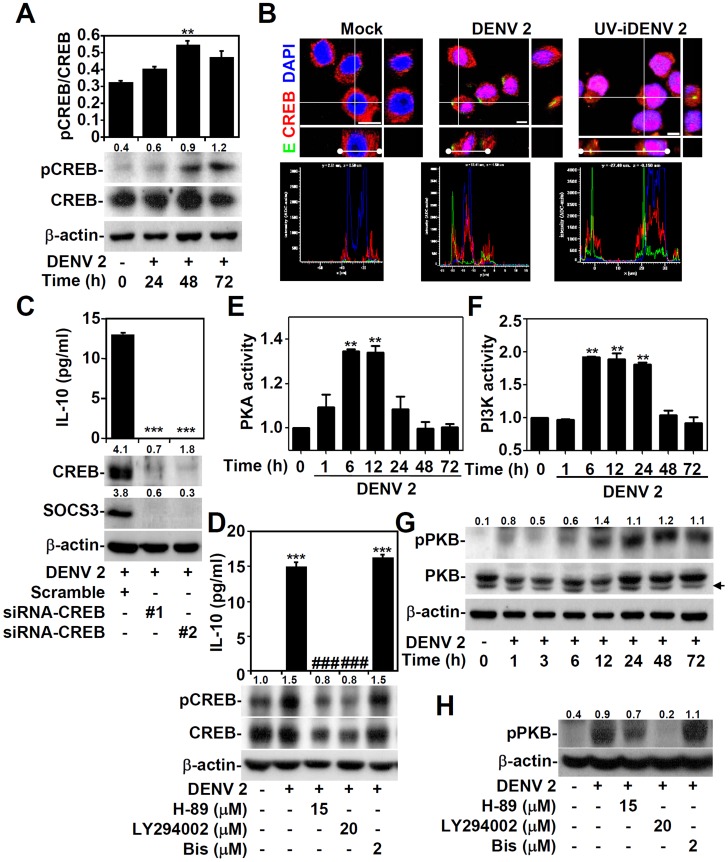
DENV infection sequentially induces CREB activation and CREB-mediated IL-10 production through PKA- and PI3K-regulated PKB. A: A Western blot showing the time-kinetic phosphorylation of CREB (pCREB Ser133) in THP-1 cells infected with DENV serotype 2 PL046 (DENV 2, MOI  = 1). ** P<0.01, compared with untreated cells. B: Immunostaining followed by confocal microscopy (with z-stacks) revealing the expression and intracellular localization of the DENV E protein (green) and CREB (red) 24 h after infection with DENV 2 (MOI  = 1) or treatment with UV-iDENV 2; DAPI (blue) was used for nuclear staining. Scale bar indicates 5 µm. Images are representative of three independent experiments and a semiquantitative analysis of CREB nuclear cytoplasmic redistribution was performed using the line profile of Leica Confocal Software. Fluorescence intensities along the white lines sectioning the cells through the cytoplasm and nucleus are plotted for DENV E, CREB, and DAPI. C: CREB expression was silenced in THP-1 cells using small interfering RNA (siRNA-CREB clone #1 and clone #2); a scrambled siRNA was used as a control. siRNA-transfected THP-1 cells were infected with DENV 2 (MOI  = 1) for 48 h. ELISA was used to quantify IL-10 production. ***P<0.001, compared with the scrambled siRNA. Western blots showing the expression of CREB and SOCS3. D: THP-1 cells were pre-treated with or without the PKA inhibitor H-89, the PI3K inhibitor LY294002, or the PKC inhibitor bisindolylmaleimide-1 (Bis) for 0.5 h, and then infected with DENV 2 (MOI  = 1) for 48 h. ELISA and Western blotting analyses were used to detect the expression of IL-10 and the indicated proteins. ***P<0.001, compared with untreated cells; ###P<0.001, compared with DENV-infected cells. PKA fluorescent assays (E) and PIP3 precipitated assays (F) were used to detect the time-kinetic activities of PKA and PI3K, respectively, in THP-1 cells infected with DENV 2 (MOI  = 1). The data are shown as fold changes with respect to the normalized values of the controls. **P<0.01, compared with untreated cells. A Western blot showing the time-kinetic phosphorylation of PKB (pPKB Ser473) in DENV 2 (MOI  = 1)-infected THP-1 cells (G) and in THP-1 cells pre-treated with or without H-89, LY294002, or Bis for 0.5 h, and then infected with DENV 2 (MOI  = 1) for 48 h (H). For the Western blotting analyses, the arrow indicates a non-specific binding signal, and β-actin was the internal control. One set of representative data obtained from three independent experiments is shown. The ratios of phosphorylated CREB and PKB to total proteins and the relative densities of CREB and SOCS3 are shown. For all experiments, the quantitative data shown represent mean ± SD values of three independent experiments.

Although we determined that DENV infection and/or attachment induces IL-10 production, the molecular mechanisms underlying the IL-10 expression were unclear. IL-10 production can be regulated by a variety of transcription factors [42]. We therefore investigated the activity of one of these in DENV-infected THP-1 cells: CREB, a transcriptional factor that activates IL-10 expression [43]–[45]. The quantitative data of Western blotting demonstrated that DENV infection significantly (*P*<0.01) activated CREB by inducing Ser133 phosphorylation in a time-dependent manner (Figure 2A). Immunostaining for expression of the DENV E and CREB proteins revealed that CREB translocated to the nucleus following DENV infection or stimulation by ultraviolet-inactivated DENV (Figure 2B). Plasmids expressing small interfering RNAs (siRNAs) specific to CREB (siCREB) were used to silence CREB expression in THP-1 cells, and this knockdown of CREB significantly (*P*<0.001) decreased DENV-induced IL-10 production (Figure 2C, upper panel), accompanied by SOCS3 down-regulation (Figure 2C, lower panel). CREB phosphorylation is mediated by PKA, PI3K/PKB, and PKC [42]. Treatment with both the PKA inhibitor H-89, which selectively inhibits only PKA, and the PI3K inhibitor LY294002 significantly (*P<*0.001) decreased DENV-induced IL-10 production (Figure 2D, upper panel) and efficiently reduced CREB phosphorylation (Figure 2D, lower panel); treatment with the broadly acting PKC inhibitor bisindolylmaleimide-1 (Bis) had no effect. Furthermore, inhibiting PKC by using myristoylated PKC inhibitor also did not cause a decrease in the DENV-induced IL-10 (Figure S2 in Text S1). Next, we investigated the potential regulation of PI3K/PKB by PKA, which has been demonstrated previously [46]. Using activity assays, we determined the time courses of PKA (Figure 2E) and PI3K (Figure 2F) activation, revealing an early activation of PKA by 1 h post-infection. Western blotting confirmed that DENV infection induces PKB phosphorylation at Ser473 (Figure 2G). Notably, pharmacologically inhibiting PKA and PI3K, but not PKC, differentially inhibited DENV-induced PKB phosphorylation (Figure 2H), suggesting that PKA, at least in part, acts upstream of PI3K/PKB. These results demonstrate that DENV infection activates PKA, PI3K/PKB, and CREB in a sequential manner, leading to IL-10 production in monocytes.

### DENV infection induces PKA/PI3K/PKB-regulated glycogen synthase kinase (GSK)-3β inactivation to facilitate CREB-mediated IL-10 production

**Figure 3:**
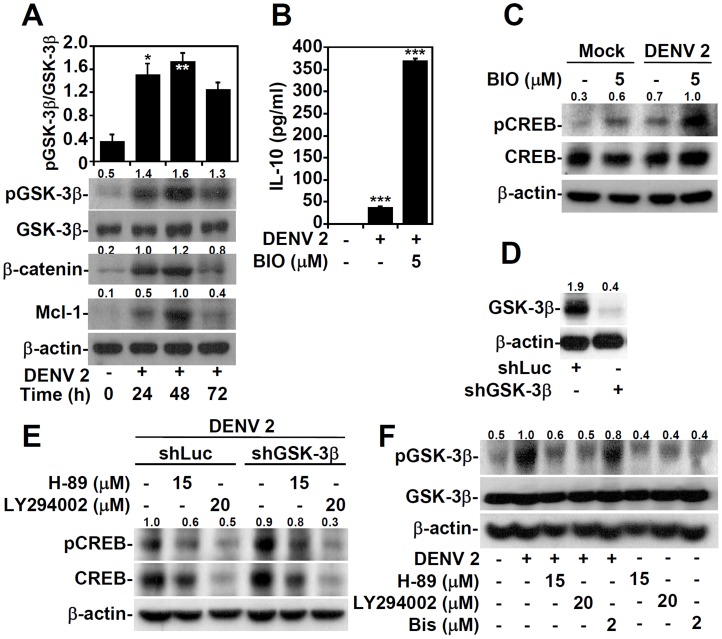
DENV infection induces PKA/PI3K-regulated GSK-3β inactivation, which facilitates CREB-mediated IL-10 production. A: A Western blot showing the time-kinetic phosphorylation of GSK-3β (pGSK-3β Ser9) and the expression of GSK-3β, β-catenin, and Mcl-1 in THP-1 cells infected with DENV serotype 2 PL046 (DENV 2, MOI  = 1). ** P<0.01 and *** P<0.001, compared with untreated cells. THP-1 cells were pre-treated with or without the GSK-3 inhibitor BIO, and then infected with DENV 2 (MOI  = 1) for 48 h. ELISA (B) and Western blotting (C) analyses were used to detect IL-10 production and CREB phosphorylation (pCREB Ser133), respectively. For the ELISA analyses, the data shown represent mean ± SD values of three independent experiments. ***P<0.001, compared with untreated cells; ###P<0.001, compared with DENV-infected cells. Western blots showing the expression of the indicated proteins in THP-1 cells transfected with lentiviral-based shGSK-3β (D) shRNA-transfected THP-1 cells pre-treated with or without H-89 or LY294002 for 0.5 h, and then infected with DENV 2 (MOI  = 1) for 48 h (E) and THP-1 cells pre-treated with or without H-89, LY294002, or Bis for 0.5 h, and then infected with DENV 2 (MOI  = 1) for 48 h (F). shLuc was used as a negative control. For Western blotting results, β-actin was the internal control. One set of representative data obtained from three independent experiments is shown. The ratios of phosphorylated GSK-3β and CREB to total proteins and the relative densities of β-catenin and Mcl-1 are shown. For all experiments, the quantitative data shown represent mean ± SD values of three independent experiments.

In addition to PKA and PI3K/PKB, CREB is also regulated by GSK-3β, which decreases the stability of CREB by phosphorylating CREB at Ser129 [47]. Furthermore, GSK-3β function – which is controlled by the PKA [48] and PI3K/PKB signaling pathways [49] – is important for CREB activity [50] and IL-10 production [51], [52]. Therefore, we hypothesized that GSK-3β may be inactivated during DENV-induced IL-10 production. Indeed, the quantitative data of Western blotting showed that GSK-3β was significantly (*P*<0.01) inhibited by phosphorylation at Ser9 [53] in DENV-infected THP-1 cells (Figure 3A, upper panel); this GSK-3β inhibition was also accompanied by an accumulation of β-catenin and Mcl-1 protein, substrates that are negatively regulated by GSK-3β [54], [55] (Figure 3A, lower panel). Furthermore, treating cells with the GSK-3 inhibitor BIO significantly (*P<*0.001) enhanced DENV-induced IL-10 production (Figure 3B) and effectively increased CREB phosphorylation at Ser133 (Figure 3C). shRNA-based GSK-3β silencing (Figure 3D) caused an increase in CREB phosphorylation at Ser133, and pharmacological inhibition of PKA and PI3K resulted in CREB dephosphorylation (Figure 3E). These findings indicate an important but not strictly necessary role for GSK-3β in regulating CREB activity during DENV infection. In addition, the DENV-induced inactivation of GSK-3β was found to be regulated by both PKA and PI3K/PKB (Figure 3F). Taken together, these data indicate that following DENV infection, both PKA and PI3K/PKB inhibit GSK-3β activity to coordinately facilitate CREB-mediated IL-10 production in monocytes.

### DENV-induced IL-10 production is regulated by spleen tyrosine kinase (Syk) signaling

**Figure 4:**
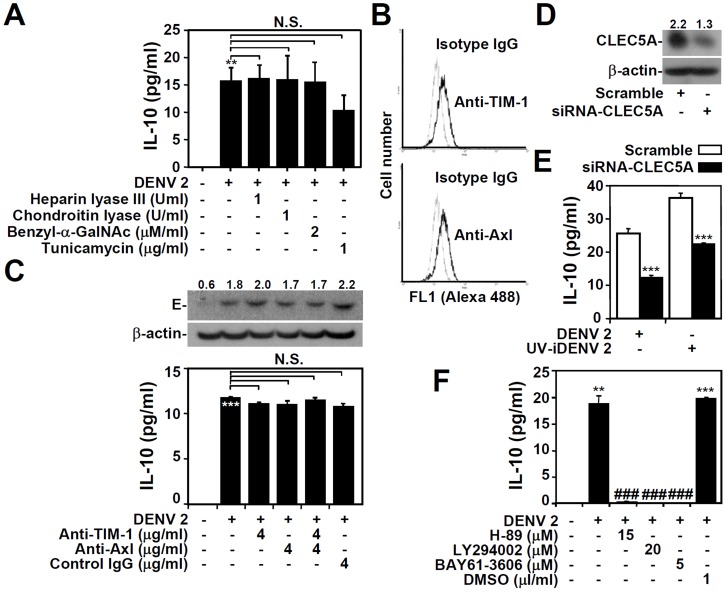
CLEC5A and Syk signaling differentially regulate DENV-induced IL-10 production. A: An ELISA showing IL-10 expression 48 h after infection of THP-1 cells pre-treated with or without heparin lyase III and chondroitin lyase for 1 h and benzyl-α-GalNAc and tunicamycin for 24 h, and then infected with DENV serotype 2 PL046 (DENV 2, MOI  = 1). B: A representative histogram of cytometric analyses of immunostaining, obtained from three independent experiments, showing expression of TIM-1 and Axl in THP-1 cells. The light gray line represents the isotype control. C: An ELISA showing IL-10 expression 48 h after infection in THP-1 cells pre-treated with or without neutralizing Abs against TIM-1 and Ax1 for 1 h, and then infected with DENV 2 (MOI  = 1) for 48 h. A Western blot showing the expression of DENV E protein. D: Western blot showing CLEC5A expression in THP-1 cells transfected with siCLEC5A; a scrambled siRNA was used as a negative control. E: Transfected THP-1 cells were infected with DENV 2 (MOI  = 1) or UV-iDENV for 48 h, and ELISA was performed to detect IL-10 production. F: An ELISA showing IL-10 expression in THP-1 cells pre-treated with or without H-89, LY294002, or Syk inhibitor BAY61-3606 for 0.5 h, and then infected with DENV 2 (MOI  = 1) for 48 h. DMSO was used for the negative control. For the ELISA analyses, the data shown represent mean values ± SD from three independent experiments. **P<0.01 and ***P<0.001, compared with untreated or control cells. ###P<0.001, compared with DENV-infected cells. N.S., not significant. For Western blotting results, β-actin was the internal control. One set of representative data obtained from three independent experiments is shown. The relative densities of E and CLEC5A are shown.

Next, we investigated the role of host receptors in regulating DENV-induced IL-10 production. Heat-inactivated DENV failed to induce IL-10 production in THP-1 cells (Figure S3 in Text S1), whereas ultraviolet-inactivated DENV induced IL-10 normally, indicating the essential role of structural proteins for this aspect of infection. Various host receptors expressed on cell surfaces have been reported to bind the DENV E protein [14]. We used heparan lyase III to cleave the extracellular heparan sulfate, and benzyl-α-GalNAc and tunicamycin to block O- and N-linked glycosylation, respectively. Although glycosylated heparan sulfate was previously reported to function as a DENV receptor [15], neither heparan sulfate cleavage nor inhibition of glycosylation resulted in inhibition of DENV-induced IL-10 production (Figure 4A). DC-SIGN and β3-integrin are host cell receptors for DENV infection [16], [17]; however, there was no expression of these proteins on the surface of THP-1 cells (Figure S4 in Text S1). Additionally, the results of competitive assays that utilized neutralizing Abs to block these proteins confirmed the independent roles of these receptors for DENV-induced IL-10 production in THP-1 cells (Figure S5 in Text S1). The cell-surface phosphatidylserine receptors TIM-1 and Axl, which were originally identified as surface receptors for the recognition of apoptotic cells, were recently identified as potential DENV entry receptors [56]. Immunostaining, followed by flow cytometric analysis, revealed low levels of expression of TIM-1 and Axl in THP-1 cells (Figure 4B); however, inhibition of TIM-1 and Axl by neutralizing Abs did not reduce DENV entry (Figure 4C, upper panel) or DENV-induced IL-10 production (Figure 4C, lower panel). CLEC5A, a member of the C-type lectin superfamily, plays a crucial role in the DENV infection-associated cytokine response [18]. We showed that CLEC5A expression was higher in DENV-susceptible THP-1 cells than in other monocytic HL-60 and U937 cells (Figure S6 in Text S1). Furthermore, A positive relationship between CLEC5A, infectious ability, and IL-10 production was also shown. Knockdown of CLEC5A expression in THP-1 cells (Figure 4D) partly, but significantly (*P<*0.001), decreased IL-10 production following DENV infection or stimulation by UV inactivated-DENV (Figure 4E). Next, we investigated the potential regulation of CLEC5A-regulated signaling during IL-10 production. Activation of the tyrosine kinase Syk positively regulates DNX activating protein 12, the downstream adaptor protein of CLEC5A [57]; pharmacological inhibition of Syk with the selective inhibitor BAY-61-3606 significantly (*P<*0.001) reduced DENV-induced IL-10 production (Figure 4F). These results demonstrate a novel role for Syk signaling in DENV-induced IL-10 production in monocytes, most likely in a CLEC5A-regulated manner.

### ADE of DENV infection amplifies IL-10 production through Syk-regulated PI3K/PKB/GSK-3β/CREB signaling

**Figure 5:**
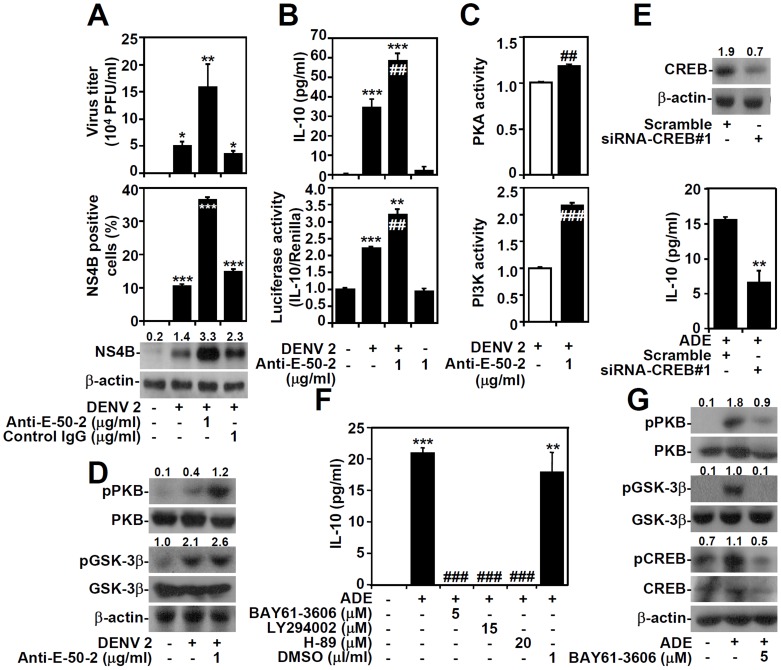
ADE intrinsically facilitates DENV-induced IL-10 expression by modulating PKA/PI3K/PKB/GSK-3β/CREB signaling in a Syk-regulated manner. THP-1 cells were infected with DENV serotype 2 PL046 (DENV 2, MOI  = 1) in the presence or absence of anti-E (clone 50–2) mAb; isotype control IgG was used for the negative control. Plaque assay, Western blotting (A and D), ELISA, luciferase reporter (B), and activity assays (C) showing the expression of the indicated proteins (48 h-post-infection), the production (48 h-post-infection) and transcriptional activation (24 h-post-infection) of IL-10, the replication of DENV 2, and the activation of PKA and PI3K (24 h-post-infection). Plaque assays and immunostaining-based flow cytometric analysis were used to quantify viral replication and NS4B expression, respectively. E: An ELISA showing IL-10 expression in DENV 2-infected CREB-silenced THP-1 cells in the presence or absence of anti-E mAb (1 µg/ml) for 48 h, called ADE; a scrambled siRNA was used as a control. A Western blot shows the expression of CREB protein. F: ELISA showing IL-10 expression in THP-1 cells pre-treated with or without H-89, LY294002, or BAY61-3606 for 0.5 h, and then infected with ADE of DENV 2 for 48 h. G: Western blots showing the expression of phosphorylated PKB (pPKB Ser473), GSK-3β (pGSK-3β Ser9), and CREB (pCREB Ser133) in THP-1 cells pre-treated with or without BAY61-3606 for 0.5 h, and then infected with ADE of DENV 2 for 48 h. For IL-10 production, the IL-10 activity assay, and viral replication, the data are shown as the mean ± SD values from three independent experiments. For the luciferase reporter assays, the data shown are the ratios of the levels of IL-10 to Renilla. *P<0.05, **P<0.01, and ***P<0.001, compared with untreated or control cells. ##P<0.01 and ###P<0.001, compared with DENV-infected cells. For Western blotting results, β-actin was the internal control. One set of representative data obtained from three independent experiments is shown. The ratios of phosphorylated PKB, GSK-3β, and CREB to total proteins and the relative densities of NS4B and CREB are shown.

Experiments have demonstrated that ADE not only facilitates DENV entry through the Fcγ receptor but also increases DENV-induced IL-10 expression [20], [32]–[34]. To investigate the regulation of IL-10 production during ADE of DENV infection, anti-E (clone 50–2) monoclonal (m) Abs were used to induce ADE, as described in a previous study [38]. A plaque assay confirmed the enhanced infection of DENV by ADE (*P<*0.05; Figure 5A, upper panel), and immunostaining-based flow cytometric analysis (*P<*0.001; Figure 5A, middle panel) and Western blotting (Figure 5A, lower panel) confirmed the increased levels of viral NS4B expression in THP-1 cells following ADE of DENV infection. As compared with DENV infection alone, the effects of ADE were further evidenced by increased IL-10 production (*P<*0.01; Figure 5B, upper panel) and transcriptional activation of IL-10 (*P<*0.01; Figure 5B, lower panel). Treating anti-E (clone 50–2) alone also did not cause IL-10 production. Using activity assays, we confirmed that ADE significantly increased DENV-activated PKA (*P<*0.01; Figure 5C, upper panel) and PI3K activity (*P<*0.001; Figure 5C, lower panel). Furthermore, Western blotting demonstrated increased phosphorylation of PKB at Ser473 and GSK-3β at Ser9 in THP-1 cells under ADE of DENV infection (Figure 5D). Consistent with DENV infection alone, silencing of CREB expression (Figure 5E, left panel) significantly (*P<*0.01) reduced IL-10 production following ADE (Figure 5E, right panel). Notably, Syk is also required for Fcγ receptor signaling and may be involved in IL-10 regulation [20]. Pharmacological inhibition of Syk, PI3K, and PKA significantly (*P<*0.001) reduced IL-10 production following ADE of DENV infection (Figure 5F). Furthermore, the Syk inhibitor BAY-61-360 also decreased ADE-induced phosphorylation of PKB at Ser473, GSK-3β at Ser9, and CREB at Ser133 (Figure 5G). These results demonstrate that Syk regulates the PI3K/PKB/GSK-3β/CREB pathway during ADE-induced IL-10 production in monocytes.

### Viral load, not serotype, determines IL-10 production levels

**Figure 6:**
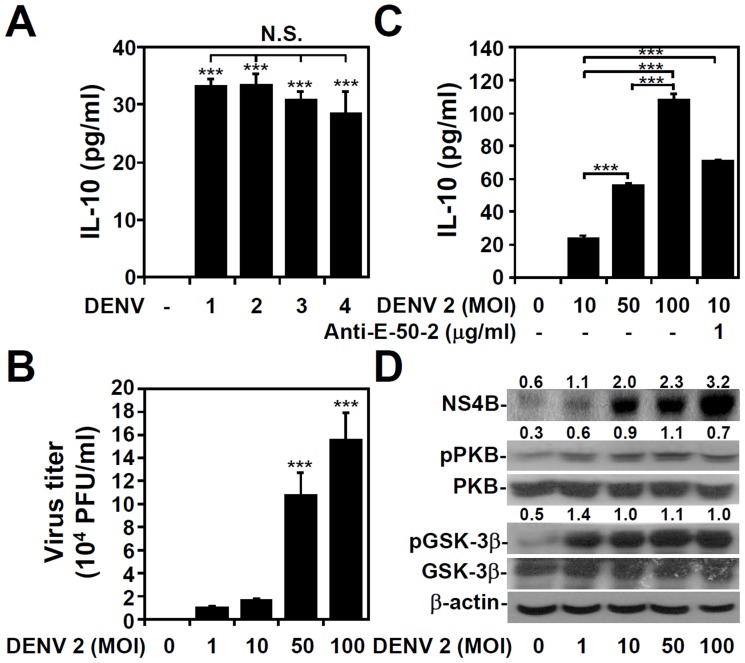
Viral load, but not serotype, affects DENV-induced IL-10 production. An ELISA showing IL-10 expression in THP-1 cells infected with DENV serotypes 1–4 (MOI  = 1) for 48 h (A) and with DENV serotype 2 PL046 (DENV 2) at the indicated MOI for 48 h in the presence or absence of anti-E (clone 50–2) mAb (C). The data are shown as the mean ± SD values from three independent experiments. ***P<0.001. N.S., not significant. B: A plaque assays showed viral replication in a MOI-dependent manner. The data are shown as the mean ± SD values from three independent experiments. *** P<0.001, compared with untreated cells. D: Western blots showing the expression of the DENV NS4B proteins, phosphorylation of PKB (pPKB Ser473), PKB, phosphorylation of GSK-3β (pGSK-3β Ser9), and GSK-3β in THP-1 cells infected with the indicated MOI of DENV 2 for 48 h. β-actin was the internal control. One set of representative data obtained from three independent experiments is shown. The ratios of phosphorylated PKB and GSK-3β to total proteins and the relative densities of NS4B are shown.

No significant differences in the ability of the four DENV serotypes to induce IL-10 production following DENV infection in THP-1 cells were observed between the four serotypes (Figure 6A). Although intrinsic ADE has been hypothesized to facilitate IL-10 production, most likely through intracellular signaling of the Fcγ receptor II [20], [32]–[34], the virus-cell interaction is also enhanced extrinsically in the canonical ADE pathway by increasing the infection rate in Fcγ receptor-bearing cells. Next, the effects of different DENV viral loads on IL-10 induction and signal regulation were investigated. Notably, a high viral load of DENV infection alone, as demonstrated by plaque assays (*P<*0.001; Figure 6B), significantly (*P<*0.001) induced IL-10 production (Figure 6C) in a multiplicity of infection (MOI)-dependent manner, although phosphorylation of PKB at Ser473 and GSK-3β at Ser9 was not increased (Figure 6D). Notably, the results of IL-10 production in THP-1 cells infected with DENV alone under a high MOI condition or with ADE infection of DENV under a lower MOI were similar. These results indicate that viral load may affect DENV-induced IL-10 production in monocytes, independent of serotype.

### DENV-induced IL-10 production facilitates virus replication

**Figure 7:**
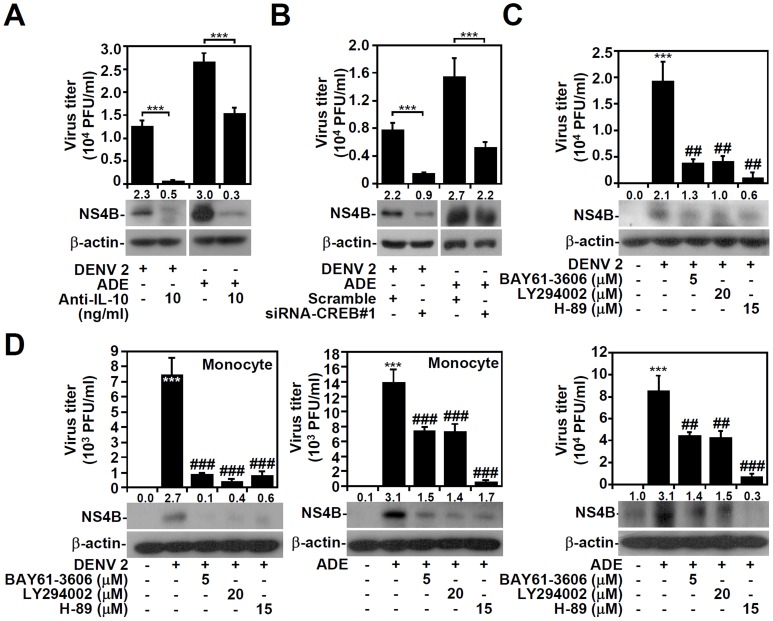
Regulation of IL-10 affects DENV replication. A plaque assay and Western blots showing viral replication and the expression of DENV NS4B protein, respectively, in THP-1 cells infected with DENV serotype 2 (DENV 2, MOI  = 1) with or without anti-E-50-2 mAb (ADE) in the presence of neutralizing anti-IL-10 Ab for 48 h (A), CREB-silenced THP-1 cells with or without ADE of DENV (MOI  = 1) infection for 48 h (B), THP-1 cells pre-treated with or without BAY61-3606, LY294002, or H-89 for 0.5 h, and then infected with DENV 2 (MOI  = 1) with or without ADE for 48 h (C) and human peripheral blood monocytes pre-treated with or without BAY61-3606, LY294002, or H-89 for 0.5 h, and then infected with or without ADE of DENV 2 (MOI  = 1) for 48 h (D). A scrambled siRNA was used as a control. β-actin was the internal control. One set of representative data obtained from three independent experiments is shown. The relative densities of NS4B are shown. For viral replication analysis, the data are shown as the mean ± SD values from three independent experiments. *** P<0.001, compared with untreated or control cells. ##P<0.01 and ###P<0.001, compared with DENV-infected cells.

IL-10 serum levels are higher in patients with DHF/DSS [25]–[31], and furthermore, ADE-enhanced IL-10/SOCS3 expression may interfere with the antiviral response to IFN [20], . Based on our earlier findings, we next investigated the importance of IL-10 signaling for DENV replication. With or without ADE, replication of DENV in THP-1 cells, as determined by plaque assays (*P<*0.001; Figure 7A, upper panel) and Western blotting of NS4B expression (Figure 7A, lower panel), was inhibited by the presence of neutralizing IL-10 Abs. With or without ADE, DENV replication, as determined by plaque assays and Western blotting of NS4B expression, was completely blocked by genetically silencing CREB (*P<*0.001; Figure 7B) and pharmacologically inhibiting Syk, PI3K, and PKA (*P<*0.01; Figure 7C). Consistent with these findings, inhibition of Syk, PI3K, and PKA also reduced DENV replication, as determined by plaque assays and Western blotting of NS4B expression, in human peripheral blood monocytes (*P<*0.001; Figure 7D). These inhibitors did not cause cell cytotoxicity (Figure S7 in Text S1). These results show that IL-10 facilitates DENV replication and that altering IL-10 regulation can affect viral replication in monocytes.

## Discussion

**Figure 8:**
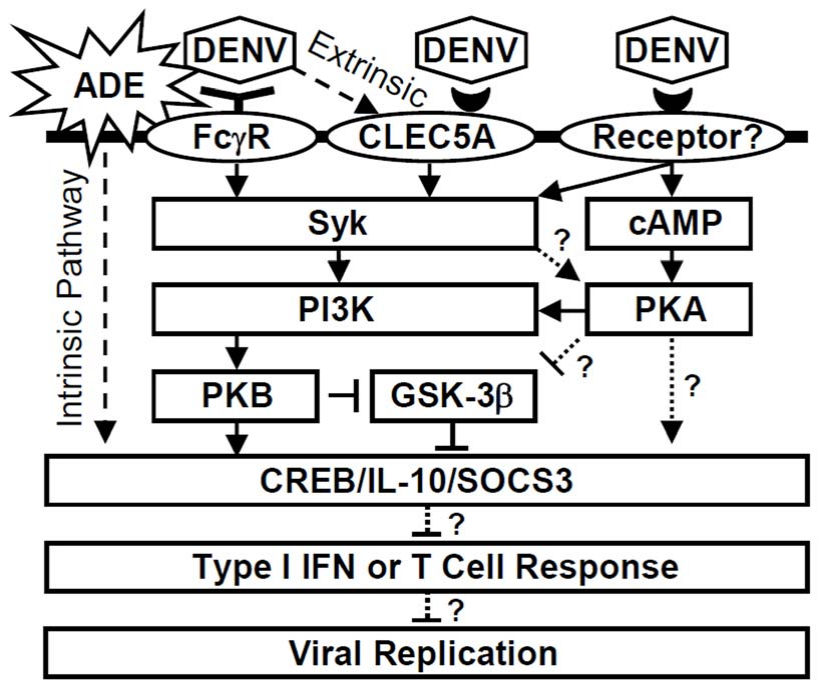
Dual hypothetical models for IL-10 regulation during ADE of DENV-infected monocytes.

Both the physiological and the pathogenic roles of IL-10 are immunosupressive. IL-10 not only suppresses inflammation during immune resolution but also affects pathogen clearance and helps alleviate immunopathology [58]. In particular, during microbial infection, IL-10 plays an essential role in relieving IFN-γ- and TNF-α-mediated immunopathology [59], [60] as well as IFNs-mediated antiviral responses. In addition to the canonical extrinsic ADE pathway, which facilitates DENV virus-cell interactions, an intrinsic ADE pathway exists that may play a role during persistent viral infections by inducing IL-10-mediated immune suppression, particularly on IFNs responses [20], [32]–[34]. ADE of DENV infection requires the Fcγ receptor [34], but the molecular regulation of Fcγ receptor signaling for IL-10 expression remains unclear; therefore, we chose to further investigate this pathway in monocytes. As summarized in Figure 8, combined with the previous studies which ADE facilitates IL-10 production [20], [32]–[34], we speculated that ADE of DENV infection and/or attachment induces not only the Fcγ receptor/Syk-facilitated (i.e., intrinsic) pathway but also the Fcγ receptor/CLEC5A partly/Syk-mediated (i.e., extrinsic) pathway, which both lead to PKA/PI3K/PKB activation, followed by CREB-mediated IL-10 production. In this study, we did not exclude the involvement of CLEC5A/Syk signaling for the intrinsic pathway of IL-10 production under ADE. In addition, we demonstrated that PKB phosphorylates GSK-3β and decreases its activity, enhancing CREB stability and inducing IL-10 production, consistent with previous studies [43]–[45], [51], [52]. However, the regulation of PKA by DENV receptors and effectors requires further investigation. Following IL-10 induction, SOCS3 is upregulated and may facilitate attenuation of T cell responses [36] and induction of IFN resistance to enhance viral replication [20], [32]–[34]. By interfering nitric oxide generation, which is regulated by STAT1/IRF1 signaling and confers anti-DENV activity, IL-10 may cause immunosuppression through SOCS3 expression [32], [34]. Based on our findings, IL-10 appears to play a permissive role with respect to DENV pathogenesis, and regulating IL-10 production may therefore provide cellular protection against DENV infection, even under ADE conditions. However, this hypothesis needs to be approved *in vivo* by using an appropriate animal model.

Following DENV infection, host cells secrete a variety of immune mediators to mediate anti-viral responses and inflammatory activation. Severe cases of DHF/DSS caused by DENV infection are clearly the result of interactions between viral and host responses. Interest in IL-10 is increasing for a variety of reasons. First, IL-10 may be a useful prognostic tool because IL-10 serum levels are positively correlated with dengue disease severity, particularly in DHF/DSS patients [25]–[31], and because IL-10 displays immunosuppressive properties during DENV infection [20], [32]–[34]. The role of the expression and activation of CD25-positive regulatory T cells, as well as relevant genetic polymorphisms, in IL-10 overproduction were previously evaluated [61], [62]; however, these studies did not provide a strong link between IL-10 and DHF/DSS progression, particularly in the context of ADE. Therefore, several possibilities remain: ADE causes IL-10 overexpression, IL-10 production facilitates ADE, or both processes are synergistically coupled. Furthermore, it is also possible that aberrant production of IL-10 could be the result of intrinsic regulation by ADE of DENV infection [32], [34]. Based on this hypothesis, the IL-10 response elicited by ADE may increase resistance to antiviral IFN-mediated immune surveillance. This study therefore favorably explores the pathogenic regulation of IL-10 signaling in ADE infection of DENV. However, the expression and the role of IL-10 can be pathologically as well as physiologically. Although clinical studies have shown that the increased levels of IL-10 can be detected and show impact on dengue disease statement. It should not be exclude that the increased levels of IL-10 may be the result of a regulatory effect more than pathogenic during later phase of the disease progression.

To escape immune surveillance, the DENV can induce several immunosuppressive mediators through unknown mechanisms, including oncostatin M (OSM; an activator of SOCS3 expression), protein inhibitor of activated STAT1 (PIAS1; a negative regulator of STAT1), SOCS1, and SOCS3 [34]. SOCS3 plays a key role downstream of IL-10 signaling in modulating the immune response [63]. With respect to IFN resistance, SOCS3 binds to IFGR1 to inhibit STAT1 interactions, and numerous human viruses, such as hepatitis C virus (HCV), human immunodeficiency virus, and Epstein-Barr virus, can facilitate SOCS3 expression [64]–[67]. Increased serum IL-10 levels as well, as increased SOCS3 expression in PBMCs, have been demonstrated previously in severe DHF/DSS patients [32], [34]. Furthermore, DENV-induced IL-10 may interfere the production of IFN-γ in T cell activation [36]. These findings confirm that IL-10 is important for DENV infection and replication. Although IL-10 may act upstream of SOCS3 in particular, a variety of transcription factors are involved in IL-10 production, including CREB, NF-κB, and MAF [68]. Regarding the early detection of IL-10 at 24 h post-infection, the factors which show crosstalk with CREB are speculated to be important for regulating IL-10 expression. In this study, we observed that DENV infection significantly activates CREB and that CREB silencing reduces DENV replication. Importantly, CREB is a transcription factor involved in the regulation of glucose homeostasis, growth factor-dependent cell survival, and immune modulation [42]. Furthermore, a pathogenic role for CREB has been demonstrated during HBV pathogenesis; the HBV X protein activates viral gene transcription by interacting with CREB, leading to activation of the HBV enhancer I [69]. CREB can also induce expression of early growth response-1, which facilitates herpes simplex virus-1 replication [70]–[72]. In addition to its role in IL-10/SOCS3-mediated immunosuppression, we hypothesize that CREB plays a role in transcriptional regulation during DENV infection and replication, which requires further investigation.

We demonstrated that DENV infection induces PKA/PI3K/PKB-mediated CREB phosphorylation, followed by IL-10 production. However, PKC was not required for these effects, even though PKC has been speculated to act downstream of Fcγ-receptor signaling during ADE of DENV infection [20], [33]. TLR3 recognizes the dsRNA form of the replicated DENV virus [73]. Following stimulation with UV-inactivated DENV, THP-1 cells still produced IL-10, suggesting the existence of IL-10 production mechanisms that are independent of viral replication. As heat-inactivated DENV did not induce IL-10 production (Figure S3 in Text S1), this suggests that the viral E protein is essential for IL-10 signaling during DENV infection. A previous study reported that the DENV E protein activates PKA through an unknown mechanism [74]. PKA is a cAMP-dependent kinase, and the activation of guanine nucleotide binding protein-coupled receptors increases intracellular cAMP levels to facilitate PKA-mediated CREB activation [75]. Therefore, other receptors and effectors of DENV-activated cAMP/PKA activation should be investigated in future studies. Signaling by integrins, which are potential receptors for DENV binding and entry, typically induces PKA-mediated CREB activation following an increase in intracellular cAMP levels [76]. However, we demonstrated that blocking integrins did not inhibit DENV-induced IL-10 production, and THP-1 cells did not express β1 and β3 integrins. We also eliminated DC-SIGN and heparan sulfate as mediators of IL-10 induction. A study of influenza A virus-infected cells demonstrated that cyclooxygenase (COX)-2-mediated prostaglandin E2 (PGE2) expression facilitates cAMP/PKA/CREB activation [77], and indeed, PGE2 receptors can rapidly increase cAMP levels [78]. Notably, DENV infection also induces COX-2 expression, followed by PGE2 production [79]. We hypothesize that DENV-induced soluble effectors may contribute to the activation of cAMP/PKA/CREB/IL-10 signaling.

In addition to ADE of DENV infection, both the Fcγ receptor and CLEC5A trigger Syk activation [57], [80], [81]. Syk is required for PI3K/PKB activation [82], [83], and PKA may also regulate PI3K/PKB [46], but the potential regulation of PKA by Syk remains unclear. However, the intracellular domain of the Fcγ receptor is required for ADE-facilitated DENV infection, indicating that intracellular signaling is initiated by the Fcγ-receptor complex [84]. Our findings speculate that both the extrinsic (Fcγ receptor/CLEC5A partly) and intrinsic (Fcγ receptor) ADE pathways can trigger Syk activation, followed by activation of the PI3K/PKB signaling axis. However, the presented data from this study has not excluded the possibility that Syk activation is also mediated by CLEC5A even under the intrinsic ADE infection. The contribution of the Fcγ receptor and viral receptors is not clear and needs further investigation. While this finding shows an important role of Syk for DENV infection, an opposing role of Syk is proposed by its role in facilitating type I IFN response and the expression of type I IFN-stimulated genes [85]. For ADE infection, a possible regulation of FcR-activated Syk may be negatively regulated by coligation with leukocyte immunoglobulin-like receptor B1 to inhibit type I IFN response [86]. A different regulation of Syk signaling by DENV and FcR is speculated for viral infection. Furthermore, this does not excluded the possibility of synergistic activation of IL-10 regulation by these two pathways during ADE of DENV infection and/or by other receptors, which may link to Syk activation.

ADE of DENV infection may induce Fcγ receptor/Syk- and/or CLEC5A partly/Syk-mediated PI3K/PKB activation. Activated PKB phosphorylates and activates CREB at Ser133, but it also phosphorylates and inactivates GSK-3β, which stabilizes CREB (GSK-3β phosphorylates CREB at Ser129, causing CREB downregulation) [47]. Consistent with previous studies [48]–[52], we observed that DENV-induced synergistic activation of PKA and PKB inhibited GSK-3β, leading to CREB-mediated IL-10 production. In monocytes/macrophages, PKC signaling is required for FcγR-mediated endocytosis [87]. Notably, PKC-induced GSK-3β inactivation facilitates the expression of IL-10 following LPS stimulation [88]. These results are inconsistent with the regulation of GSK-3β by DENV infection identified in this study, where PKC is not required for DENV-induced GSK-3β inactivation, as well as IL-10 production. GSK-3β modulation is known to be important for cell growth, differentiation, apoptosis, and inflammatory activation [89]. Inactivation of GSK-3β increases not only the stability of CREB for IL-10 production but also enhances the activity of β-catenin and Mcl-1 for cell growth and survival [54], [55]. Hepatitis C virus NS5A protein induces β-catenin accumulation by inactivating GSK-3β [90]. During *Helicobacter pylori* infection, PKB-mediated GSK-3β inactivation plays an essential role in Wnt signaling activation and cell proliferation [91]. Following DENV-induced GSK-3β inactivation, the physiological and pathological roles of accumulated β-catenin and Mcl-1 may contribute to the pathogenesis of DENV infection and replication.

In conclusion, an excessive or poorly timed IL-10 production may allow the pathogen to escape immune surveillance during DENV pathogenesis. This study demonstrates a molecular basis for IL-10 induction during DENV infection, as well as during ADE of DENV infection in human monocytes. For strengthening the significance of our findings, patients' serum and/or humanized antibodies are suggested to be examined for verifying the consistent pathway caused by Fc receptors' affinity. Current study showed that DENV infects macrophages and causes mild IL-10 production [92]. However, DENV infection does not induce significant IL-10 release from immature myeloid dendritic cells [93]. Not only in monocytes, it is crucial to check the identified pathways in other IL-10-producing cells and/or the natural targeting cells *in vivo*. With respect to a possible pathogenic role for aberrant IL-10 production in DHF/DSS patients, targeting the Syk/PKA/PI3K/PKB/GSK-3β/CREB signaling axis may represent a viable therapeutic strategy for combating the progression of severe dengue diseases.

## Supporting Information

Text S1 **Supplementary information figures.** **Figure S1. Supernatants of C3/36 cells do not cause IL-10 production in monocytes.** THP-1 cells infected with DENV serotype 2 PL046 (DENV 2, MOI  = 1) or treated with supernatants of C6/36 cells for 48 h were assessed for IL-10 production by ELISA. The quantitative data shown represent mean ± SD values of three independent experiments. *** P<0.001, compared with untreated cells. **Figure S2. Pharmacologically inhibiting PKC does not decrease DENV-induced IL-10 production in monocytes.** THP-1 cells were pre-treated with or without the PKC inhibitor bisindolylmaleimide-1 (Bis) or myristoylated PKC inhibitor for 0.5 h, and then infected with DENV 2 (MOI  = 1) for 48 h. ELISA was used to detect the expression of IL-10. DMSO was used for the negative control. The quantitative data shown represent mean ± SD values of three independent experiments. **P<0.01 and ***P<0.001, compared with untreated cells. **Figure S3. Heat-inactivated DENV does not cause IL-10 production in monocytes.** THP-1 cells infected with alive DENV or heat-inactivated DENV (iDENV) serotype 2 PL046 (DENV 2, MOI  = 1) for 48 h were assessed for IL-10 production by ELISA. The quantitative data shown represent mean ± SD values of three independent experiments. ** P<0.01, compared with untreated cells. **Figure S4. Expression of β1-integrin, β3-integrin, and DC-SIGN in monocytes.** Representative histogram of immunostaining-based flow cytometric analysis determined the expression of β1-integrin, β3-integrin, and DC-SIGN in THP-1 cells. Staining of secondary antibody and isotype control IgG was used for the background controls. **Figure S5. Neutralizing DC-SIGN and β3-integrin does not decrease DENV-induced IL-10 production in monocytes.** THP-1 cells were pre-treated with or without the neutralizing antibodies against DC-SIGN and β3-integrin for 0.5 h, and then infected with DENV 2 (MOI  = 1) for 48 h. ELISA was used to detect the expression of IL-10. The quantitative data shown represent mean ± SD values of three independent experiments. ***P<0.001, compared with untreated cells. ns, not significant. **Figure S6. The relationship between the expression of CLEC5A, viral protein, and IL-10 in monocytes.** Immunostaining-based flow cytometric analysis (A and B) and ELISA analyses were used to detect the expression of CLEC5A, DENV NS4B, and IL-10 in THP-1, HL-60, and U937 cells without or with DENV 2 (MOI  = 1) infection for 48 h. The data shown represent mean ± SD values of three independent experiments. **P<0.01 and ***P<0.001, compared with THP-1. **Figure S7. Treatment of inhibitors of Syk, PI3K, and PKA does not cause cytotoxicity in DENV-infected monocytes under ADE.** THP-1 cells and purified human monocytes were pre-treated with or without the Syk inhibitor BAY61-3606, PI3K inhibitor LY294002, and PKA inhibitor H-89 for 0.5 h, and then infected with DENV 2 (MOI  = 1) with or without ADE for 48 h. LDH release was used to detect the induction of cytotoxicity. The relative data, as compared with control, shown represent mean ± SD values of three independent experiments. ns, not significant.
(PDF)
