# Supplementary material for: Antibody-Dependent Enhancement Infection Facilitates Dengue Virus-Regulated Signaling of IL-10 Production in Monocytes
Source: PLoS Negl Trop Dis. 2014 Nov 20;8(11):e3320. doi: 10.1371/journal.pntd.0003320 (PMC4239119; doi:10.1371/journal.pntd.0003320)
Supplement: Text S1 — Supplementary information figures. Figure S1. Supernatants of C3/36 cells do not cause IL-10 production in monocytes. THP-1 cells infected with DENV serotype 2 PL046 (DENV 2, MOI = 1) or treated with supernatants of C6/36 cells for 48 h were assessed for IL-10 production by ELISA. The quantitative data shown represent mean ± SD values of three independent experiments. *** P<0.001, compared with untreated cells. Figure S2. Pharmacologically inhibiting PKC does not decrease DENV-induced IL-10 production in monocytes. THP-1 cells were pre-treated with or without the PKC inhibitor bisindolylmaleimide-1 (Bis) or myristoylated PKC inhibitor for 0.5 h, and then infected with DENV 2 (MOI = 1) for 48 h. ELISA was used to detect the expression of IL-10. DMSO was used for the negative control. The quantitative data shown represent mean ± SD values of three independent experiments. **P<0.01 and ***P<0.001, compared with untreated cells. Figure S3. Heat-inactivated DENV does not cause IL-10 production in monocytes. THP-1 cells infected with alive DENV or heat-inactivated DENV (iDENV) serotype 2 PL046 (DENV 2, MOI = 1) for 48 h were assessed for IL-10 production by ELISA. The quantitative data shown represent mean ± SD values of three independent experiments. ** P<0.01, compared with untreated cells. Figure S4. Expression of β1-integrin, β3-integrin, and DC-SIGN in monocytes. Representative histogram of immunostaining-based flow cytometric analysis determined the expression of β1-integrin, β3-integrin, and DC-SIGN in THP-1 cells. Staining of secondary antibody and isotype control IgG was used for the background controls. Figure S5. Neutralizing DC-SIGN and β3-integrin does not decrease DENV-induced IL-10 production in monocytes. THP-1 cells were pre-treated with or without the neutralizing antibodies against DC-SIGN and β3-integrin for 0.5 h, and then infected with DENV 2 (MOI = 1) for 48 h. ELISA was used to detect the expression of IL-10. The quantitative data shown represent m [file pntd.0003320.s001.pdf]

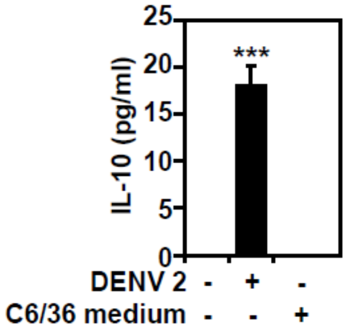

Supplementary Figure S1: Supernatants of C3/36 cells do not cause IL-10 production in monocytes. THP-1 cells infected with DENV serotype 2 PL046 (DENV 2, MOI = 1) or treated with supernatants of C6/36 cells for 48 h were assessed for IL-10 production by ELISA. The quantitative data shown represent mean ± SD values of three independent experiments. *** P < 0.001, compared with untreated cells.

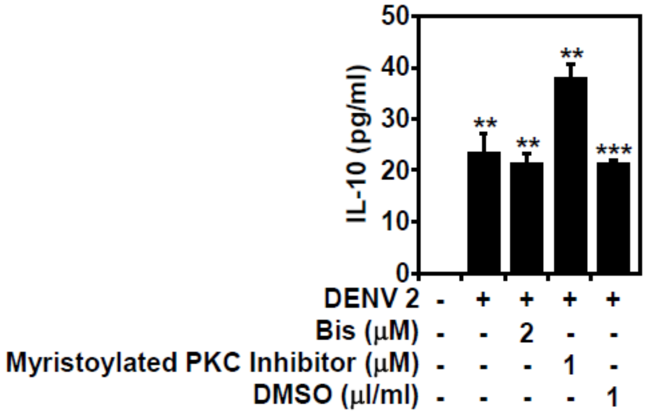

Supplementary Figure S2: Pharmacologically inhibiting PKC does not decrease DENV-induced IL-10 production in monocytes. THP-1 cells were pre-treated with or without the PKC inhibitor bisindolylmaleimide-1 (Bis) or myristoylated PKC inhibitor for 0.5 h, and then infected with DENV 2 (MOI = 1) for 48 h. ELISA was used to detect the expression of IL-10. DMSO was used for the negative control. The quantitative data shown represent mean ± SD values of three independent experiments. **P < 0.01 and ***P < 0.001, compared with untreated cells.

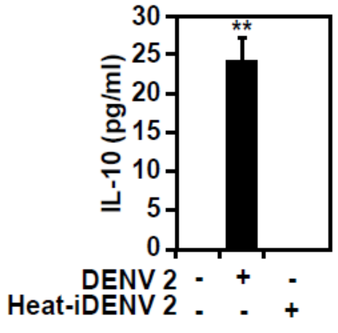

Supplementary Figure S3: Heat-inactivated DENV does not cause IL-10 production in

monocytes. THP-1 cells infected with alive DENV or heat-inactivated DENV (iDENV) serotype 2 PL046 (DENV 2, MOI = 1) for 48 h were assessed for IL-10 production by ELISA. The quantitative data shown represent mean ± SD values of three independent experiments. ** $P < 0.01$, compared with untreated cells.

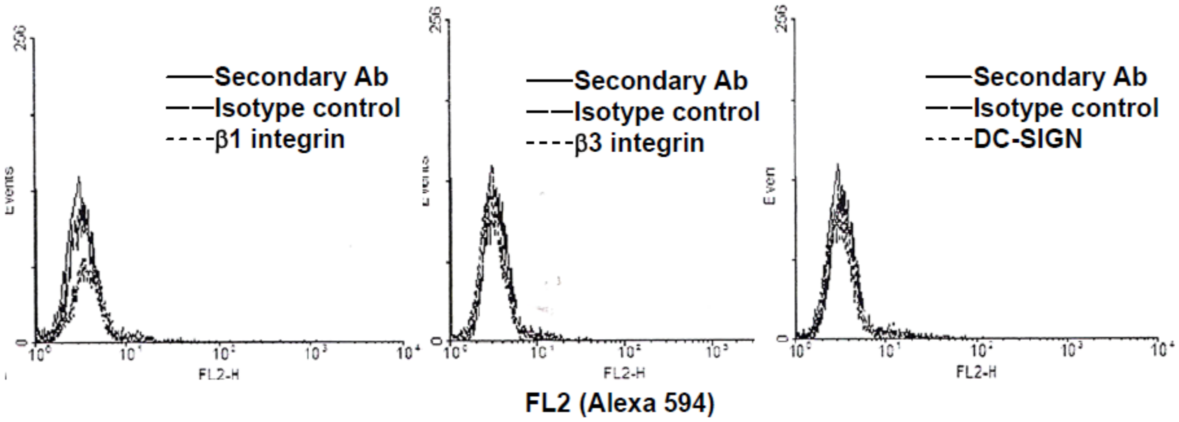

Supplementary Figure S4: Expression of β1-integrin, β3-integrin, and DC-SIGN in monocytes. Representative histogram of immunostaining-based flow cytometric analysis determined the expression of β1-integrin, β3-integrin, and DC-SIGN in THP-1 cells. Staining of secondary antibody and isotype control IgG was used for the background controls.

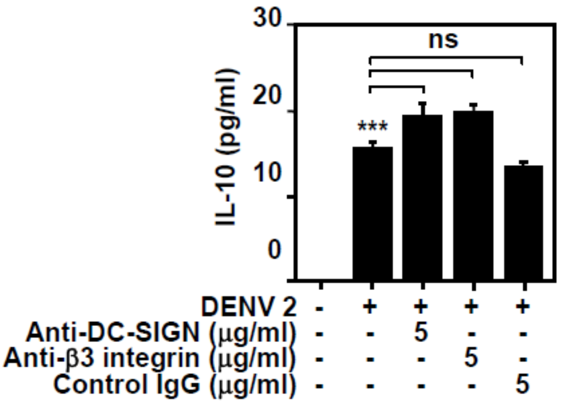

Supplementary Figure S5: Neutralizing DC-SIGN and β3-integrin does not decrease DENV-induced IL-10 production in monocytes. THP-1 cells were pre-treated with or without the neutralizing antibodies against DC-SIGN and β3-integrin for 0.5 h, and then infected with DENV 2 (MOI = 1) for 48 h. ELISA was used to detect the expression of IL-10. The quantitative data shown represent mean ± SD values of three independent experiments. ***$P < 0.001$, compared with untreated cells. ns, not significant.

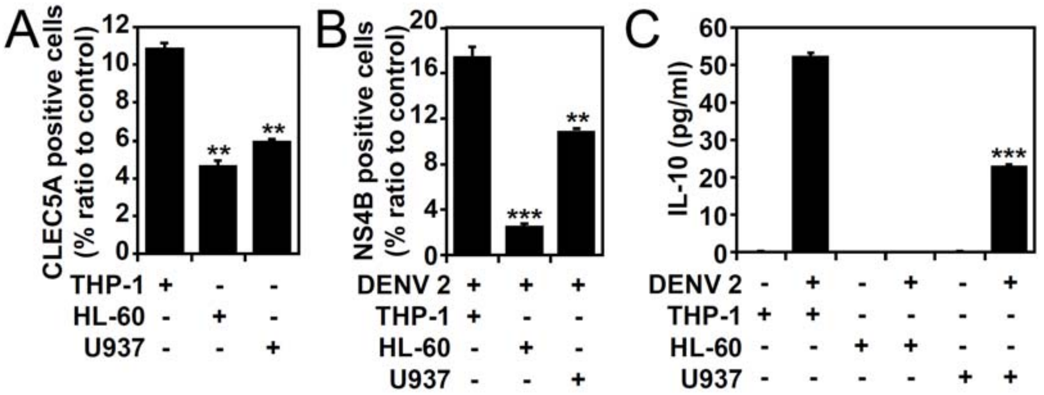

Supplementary Figure S6: The relationship between the expression of CLEC5A, viral protein, and IL-10 in monocytes. Immunostaining-based flow cytometric analysis (A and B) and ELISA analyses were used to detect the expression of CLEC5A, DENV NS4B, and IL-10 in THP-1, HL-60, and U937 cells without or with DENV 2 (MOI = 1) infection for 48 h. The data shown represent mean ± SD values of three independent experiments. **P < 0.01 and ***P < 0.001, compared with THP-1.

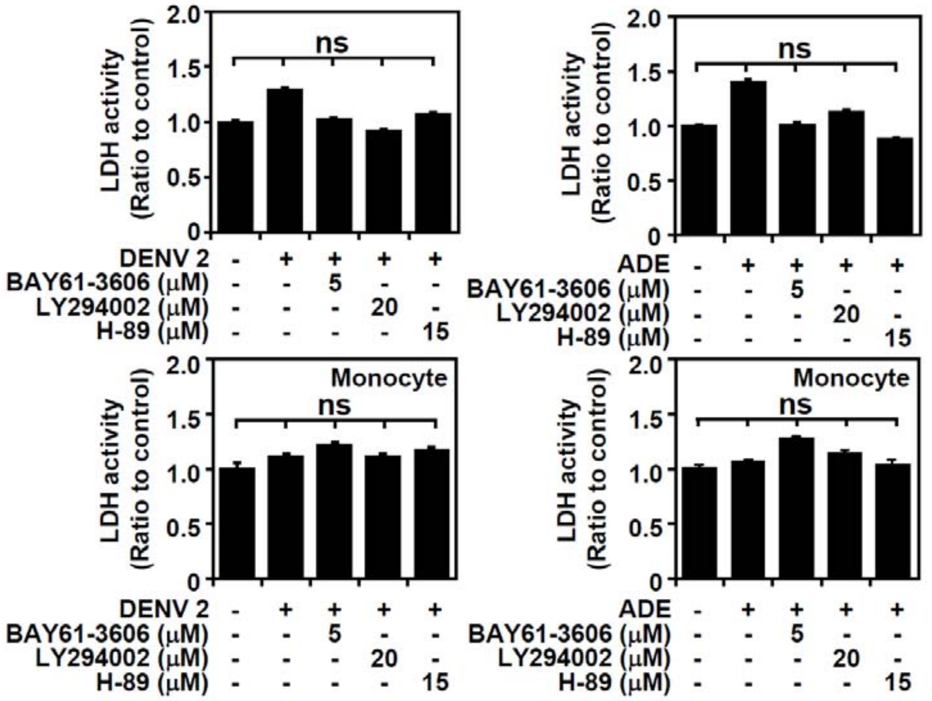

Supplementary Figure S7: Treatment of inhibitors of Syk, PI3K, and PKA does not cause cytotoxicity in DENV-infected monocytes under ADE. THP-1 cells and purified human monocytes were pre-treated with or without the Syk inhibitor BAY61-3606, PI3K inhibitor LY294002, and PKA inhibitor H-89 for 0.5 h, and then infected with DENV 2 (MOI = 1) with or without ADE for 48 h. LDH release was used to detect the induction of cytotoxicity. The relative data, as compared with control, shown represent mean ± SD values of three independent experiments. ns, not significant.
